# Measuring Temporal Change in Scrub Vegetation Cover Using UAV‐Derived Height Maps: A Case Study at Two UK Nature Reserves

**DOI:** 10.1002/ece3.70463

**Published:** 2024-10-22

**Authors:** Matthew Jordan, Jim Vafidis, Mark Steer, Kathy Fawcett, Kathy Meakin, Gareth Parry, Matthew Brown

**Affiliations:** ^1^ University of the West of England Bristol UK; ^2^ Eco‐Explore Mountain Ash UK; ^3^ Evidence Nature Dursley UK; ^4^ Forestry England Forestry Comission Yorkshire UK; ^5^ Cardiff Harbour Authority Cardiff UK

**Keywords:** photogrammetry, reserve management, scrub, temporal, UAV

## Abstract

Measuring the outcome of practical interventions and actions helps to inform conservation management objectives and assess progress towards objectives and targets. Measuring success also informs future management by identifying actions that are effective and those that are not. Scrub vegetation is an important habitat type in terrestrial ecosystems, providing important shelter and food resources for biodiversity and livestock. Much of practical land management in the UK involves the monitoring and management of scrub, and current drone‐based methods of scrub collection requires expensive equipment or complex methods. A 2021 paper determined a cheap and simple way to determine scrub levels, and this could potentially be used to map temporal changes, as well as identify directional change in scrub. This study looks at whether the method outlined in the 2021 study could be used to measure temporal and directional changes in scrub cover on two nature reserves in the UK: Daneway Banks in Gloucestershire and Flat Holm Island in the Severn Estuary. Scrub levels at Daneway Banks increased from 14.63% in 2015 to 16.52% in 2017, before decreasing to 14.89% in 2021 due to managed cutting and clearing. Scrub cover at Flatholm Island decreased from 10.18% in 2019 to 8.71% in 2021. The exact locations of scrub growth and loss for each site was also calculated and mapped. This approach was found to be a viable way of measuring temporal and directional change in scrub levels. The data can also be used to reframe changes in scrub levels as a shift towards vegetation succession or reduction, to better visualise how changes in scrub levels affect overall site management goals, and is a cheaper, more accessible alternative to current methods of measuring temporal vegetation changes.

## Graphical abstract

This study looks at temporal changes in scrub cover on two nature reserves in the UK: Daneway Banks in Gloucestershire and Flat Holm Island in the Severn Estuary. In this study, we assess how multiple datasets obtained using a photogrammetric point cloud workflow previously outlined in Vafidis et al. (Ecology and Evolution, **11**, 2021, 6789) can be used to assess scrub cover management goals and temporal changes in scrub levels at two nature reserves.

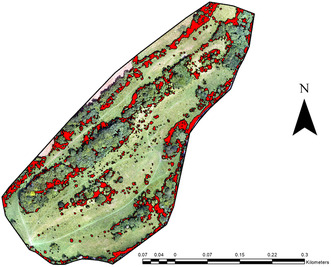

## 1 Introduction

The effective meeting of conservation objectives is a highly sought after, and ongoing goal within conservation organisations. Meeting objectives effectively allows these groups to achieve more and better preserve species and habitats. However, to do this, knowledge of the target species or habitat is required, as well as the monitoring of progress towards conservation objectives, so that management plans can be adjusted based on whether the conservation action is having the desired effect, and at what rate progress is being made towards conservation objectives (Berger‐Tal and Lahoz‐Monfort 2018). However, it is becomingly increasingly recognised that habitats and sites are highly dynamic (Zeller et al. 2020), with constant changes occurring that could affect the progress of any management goals. This requires management plans and targets to be constantly adjusted (Estes Jr. et al. 2021), which in turn requires frequent monitoring of progress to make informed conservation decisions, as well as adjust management objectives based on new information (Pullin et al. 2013; Kapos et al. 2009).

The monitoring and management of scrub vegetation is a particularly challenging aspect of site management. As an important habitat type, scrub often acts as an ecotone between woodland and more open habitat, and contains diverse communities and species encompassing a wide range of vegetation (El Balti 2021; Gimingham, Chapman, and Webb 1979). Scrub can also increase the biodiversity of a site, often having more species variety than woodland, and can act as a suitable alternative habitat if a species is displaced from its natural habitat (Keith et al. 2014; McArthur and Kitchen 2007). However, too much scrub can start encroaching on existing woodland and grassland habitats and, if not managed, the scrub can succeed into woodland, which would not be suitable habitat for many grassland and heathland species. It is important to know how scrub levels are changing on a site over time as a result of scrub management, to know whether more or less scrub management is required, as well as to ensure that current scrub management methods are having an effect. Because of this, a lot of UK site management goes into scrub control and the managing of scrub levels, and many site designations, such as sites of special scientific interest (SSSIs) and special areas of conservation (SACs) have conditions related to maintaining and managing suitable amounts of scrub on a site.

Current ground‐based assessments of scrub, are usually based on subjective assessments of scrub cover by surveyors, typically in the form of structured walks as recommended by Common Standards Monitoring Guidance (Joint Nature Conservation Committee 2019). While this does allow for a broad assessment of changes in scrub levels, it does not provide an accurate measure of change across the whole site. Depending on when along the transect the scrub assessments are made, inaccurate assessments could be made about whether scrub has increased or decreased across the site as a whole. An example of this could be the areas where the scrub assessments are carried out having less scrub, leading to a conclusion of lower scrub levels despite overall scrub levels across the site having increased. The measured levels of scrub cover are also often subjective, with the assessor making a broad estimate based on what they can see. More frequent surveys may exacerbate this problem, as a surveyor may be less likely to notice slight changes in the vegetation on‐site if they are there frequently, and increased familiarity with the site could lead to a lack of objectivity. On‐foot surveying can also be time‐consuming and costly, especially for organisations that manage large numbers of sites.

Scrub is loosely defined, encompassing a wide range of communities and compositions (El Balti 2021; Dierßen 2008), and is characterised based on its state as a transitional habitat between ground vegetation and woodland, defined as ‘all stages from the scattered bushes to closed‐canopy vegetation… usually less than 5 m tall’ (Mortimer et al. 2000). Because of this, the spatial attributes of vegetation, primarily its height, can be used to distinguish scrub from woodland and ground vegetation. Single vegetation layers, including scrub, in semi natural systems, can be isolated from each other through the categorisation of the surface layer based on its height. This approach has been used successfully when calculating the biomass of shrub‐grassland habitats (Cunliffe, Brazier, and Anderson 2016), and previous studies have used the structural component of vegetation height as a sole defining characteristic of scrub (Vafidis et al. 2021) using the typical range of 1–5 m (Mortimer et al. 2000). In this manner, the scrub in an area can be characterised and quantified by assessing the structural component of vegetation height.

Unmanned aerial vehicles (UAVs), or drones, have been identified as a potential alternative to ground‐based scrub monitoring through the use of high‐resolution imagery. Drones have been shown to provide more accurate data than traditional ground‐based methods with regards to habitat mapping and land cover analysis, primarily in the form of quantification of vegetation structure (Wich and Piel 2021; Ancin‐Murguzur et al. 2019), having higher resolution than satellite imagery (Inoue 2020) that often cannot identify small vegetation patches due to a lack of image resolution (Marston et al. 2017). While drones have been used to measure spatial and temporal changes in vegetation structure and habitat, most current studies rely on expensive equipment or complex methodologies such as LiDAR (Resop, Lehmann, and Hession 2021; Hyyppä et al. 2020), multispectral sensors (Villoslada et al. 2020) or complex machine learning (Cruz et al. 2023; Detka et al. 2023), which may be unfeasible in terms of cost or training requirements.

In 2021, a study was carried out using photogrammetric point cloud modelling of UAV‐derived imagery to identify scrub cover based on height at two sites; Daneway Banks and Flatholm Island (Vafidis et al. 2021). The study found that consumer‐grade UAVs can be used to generate structure‐from‐motion (SfM) point clouds derived from aerial images to generate 3D models at a very high resolution (Vafidis et al. 2021). This method allows accurate data on scrub levels to be collected without the use of expensive sensors or machine learning methods.

This method of scrub assessment appears to have high replicability and ease of accessibility, making it potentially of use to conservation practitioners. This method could hypothetically be used over multiple years, allowing temporal changes in scrub cover to be easily determined, as well as specific changes in small scrub stands to be identified. It is also hypothesised that this method could allow for directional change in scrub levels (i.e., whether scrub is being cleared, or succeeding into woodland) to be assessed through monitoring changes in grassland and woodland levels in addition to scrub.

In this study, we assess whether the method previously outlined in Vafidis et al. (2021) for scrub assessment, can be used to measure temporal changes in scrub, including changes in specific scrub stands, as well as identify directional change in scrub through the assessment of grassland and woodland at two UK nature reserves.

## 2 Materials and Methods

### 2.1 Aims

This study aims to use height maps generated from UAV‐derived point cloud aerial images to assess whether drone photogrammetry can be used to measure temporal and directional changes in scrub levels across two sites: Daneway Banks and Flatholm Island.

### 2.2 Study Sites

This study investigates two sites, both of which are UK nature reserves: Daneway Banks in Gloucestershire (Grid Reference SO939037) and Flatholm Island in the Severn Estuary (Grid Reference ST221649).

Daneway Banks is a 16.9‐ha reserve and is designated as an SSSI. It is comprised mainly of calcareous and neutral unimproved grassland, although a small woodland made up primarily of European Beech (*Fagus sylvatica*), Common Yew (*Taxus baccata*) and Common Whitebeam (*Sorbus aria*) runs through the centre of the site. The most common scrub species on site are Blackthorn (*Prunus spinose*), Common Hawthorn (*Crataegus monogyna*) and Dog‐rose (*Rosa canina*). Topographically, the site is a south, south‐east facing hill, with the height of the site varying from 122 m above sea level at its lowest point, to 175 m above sea level at its highest. The site is grazed by sheep and ponies from mid‐autumn to spring to keep the sward height low and is left ungrazed through spring and summer (Royal Entomological Society 2021). Manual removal of scrub is also carried out on a regular basis, with an aim to control excessive scrub levels (Natural England 2003).

Flatholm Island is a Welsh island located in the Severn estuary. The terrestrial area of the reserve is 35 ha, and the site is a designated SSSI due to the presence of several rare plant species, such as Wild Leek (*Allium ampeloprasum*) and Rock‐sea lavender (*Limonium binervosum*). The site is also home to a breeding colony of lesser black‐backed gulls (*Larus fuscus*) (Cardiff Council 2012). The site is divided into two parts, with the northern part of the island heavily managed through manual removal of weeds and grazing by a population of Soay sheep, although the population was castrated in 1998 to allow for a slow removal of the population. The southern side of the island is much less managed, with the only management being some annual coppicing of Elder (*Sambucus nigra*) and other scrub clearance, in order to maintain a diverse age structure of elder and suitable habitat for migratory birds (pers. comm).

Before any data collection took place, it was ensured that the vegetation height range of 1–5 m was suitable for including all scrub on the two sites, and excluding other vegetation. Given the needs of the site, and the definition of scrub used in this study, which is ‘all stages from the scattered bushes to closed‐canopy vegetation… usually less than 5 m tall’ (Mortimer et al. 2000), it was determined that a height of 1–5 m was a suitable metric to define scrub with regards to surveys at these two sites.

### 2.3 Data Collection

Datasets were collected via drone flights in 2015, 2017 and 2021 for Daneway Banks, and 2019 and 2021 for Flatholm. Transects were planned, created, and carried out using Pix4D Capture and all flights were pre‐programmed with 80% photo overlap so orthomosaics could be easily created from the photos. Drone sensors were calibrated before data collection and all images were saved as tagged image file format (tiff) on SD cards. The drones used for all data collection were assessed and found to be suitable in terms of detail and accuracy for the needs of the surveys (with the possible exception of the 2015 drone, as justification for its selection could not be found), and all flights took place during the day, when light conditions were judged to be suitable enough for accurate data collection. All altitudes were relative to the launch position of the drone.

#### 2.3.1 Daneway 2015 Data Collection

An unknown fixed‐wing drone was used for the 2015 flights, equipped with a LCE‐5000_E20mmF2.8_20.0_5456x3632 RGB sensor. Flights were carried out on an unknown date in 2015 at an unknown flight speed. Across a single flight, 468 images were collected, with a ground sample distance (GSD) of 4.42 cm/px.

#### 2.3.2 Daneway 2017 Data Collection

The 2017 flights were carried out using a DJI T600 Inspire 1 quadcopter drone equipped with a 12 MP Zenmuse X3 RGB sensor. Flights were carried out on the 3rd July 2017, using a front and side overlap setting of 80% at an altitude of 50 m above ground level. The drone flight speed was set to normal, approximately 5 m/s. Across five flights, 1127 images were collected, which took 78 min of flight time, with a GSD of 2.49 cm/px.

#### 2.3.3 Daneway 2021 Data Collection

The 2017 flights were carried out using a Mavic 2 Zoom quadcopter drone equipped with a 1/2.3″ CMOS sensor. Flights were carried out on the 25th May 2021, using a front and side overlap setting of 80% at an altitude of 50 m above ground level. The drone flight speed was set to normal, approximately 5.5 m/s. Across three flights, 987 images were collected, which took 41 min of flight time, with a GSD of 2.53 cm/px.

#### 2.3.4 Flatholm Data Collection

Both the 2019 and 2021 Flatholm flights were carried out using a DJI T900 Inspire 2 equipped with a 20 MP Zenmuse X4S RGB sensor. Flights were carried out on the 21st May 2019 and the 20th July 2021, using a front and side overlap setting of 80% at an altitude of 75 m above ground level in 2019, and 50 m above ground level in 2021. The drone flight speed was set to normal, approximately 5 m/s. For the 2019 flights, 1417 images were collected across seven flights, taking 145 min of flight time, with a GSD of 2.34 cm/px. For the 2021 flights, 2206 images were collected across five flights, taking 106 min of flight time, with a GSD of 1.89 cm/px.

### 2.4 Data Analysis

#### 2.4.1 Creation of Initial Scrub Cover Maps

The images were uploaded to Pix4D Mapper, which used metadata contained within the image files, including UAV coordinates, drone height above ground, and camera parameters to automatically create geo‐referenced orthomosaics of the sites, using the standard ‘3D Maps’ template within the Pix4D Mapper software. Matching points across multiple uploaded images were automatically identified and combined with 3D coordinates calculated using Structure from Motion algorithms to create a densified point cloud which was then ortho‐rectified to create an orthomosaic. Digital surface models (DSMs) containing information on the height of the terrain and all features within the flight area, including vegetation, and digital terrain models (DTMs) containing information on the ground height of the area were also automatically created using the same metadata as part of the template.

The DSMs and DTMs were exported as raster tiff files and imported into ArcGIS Pro 2.7 (Esri Ltd., 2021). The ‘Raster Calculator’ tool was used to subtract the DTM from the DSM to remove the height of the terrain from the DSM, leaving a raster that showed only the height of any non‐terrain features within the flight area, including vegetation. Polygons were then manually drawn around the site boundary and the ‘Clip Raster’ tool was used to remove anything outside of that polygon, leaving only the features within the site boundaries. For Flatholm Island, further polygons were drawn around any buildings or structures within the study boundary, and the clip raster tool used to remove them from the raster, leaving only vegetation features. No features other than vegetation were found within the Daneway raster and so this step was not performed on those datasets.

The properties of the vegetation height raster was then edited from showing a continuous height range to instead classifying all features into one of three height bands; Ground Vegetation and Other Surfaces (below 1 m), Scrub (1–5 m) and Other Vegetation (above 5 m). This classification was used based on the definition of scrub established by the JNCC (Mortimer et al. 2000) and used in the Vafidis et al. (2021) study, and was verified by visiting scrub stands on both sides and identifying the shortest scrub stands found on site, which were all found to be 1 m or above. The scrub layer was then isolated by using the ‘Setnull’ tool to convert the ground vegetation and woodland height bands to null, removing them from the dataset. The ‘Create Attribute Table’ tool was then used on the isolated scrub layer to find the total area of the site covered in scrub in square metres. This was then converted to a percentage by dividing the area covered by scrub by the total area of the site boundary and then multiplying the result by 100. To quantify temporal changes in scrub cover over time, these percentage values were then compared to each other, and the percentage increase or decrease in scrub cover between datasets was calculated.

#### 2.4.2 Ground Verification

For all datasets excluding the 2015 Daneway Banks dataset, ground verification was carried out. This ground verification included picking 20 points of scrub and 10 points of ground vegetation across each site and manually measuring them. The height of these points was then compared to the scrub cover maps, to ensure that all scrub points were included within the scrub height band, and that all points of ground vegetation were not included in the scrub height band. This ground verification included a ground vegetation point of 98.4 and 99.1 cm at Daneway banks and Flatholm Island, respectively, and a scrub point of 104 and 101.5 cm at Daneway banks and Flatholm Island, respectively. This was to ensure that even a slight consistent change in height values between the manually measured points and the drone‐derived height values would be detected in the hight bands.

#### 2.4.3 Creation of Maps Showing Temporal Changes in Scrub Cover

To identify specific patches of scrub that had grown or been lost over time, the most recent scrub layer for each dataset were subtracted from the oldest scrub layer for each dataset (the 2015 dataset for Daneway Banks and the 2019 Dataset for Flatholm Island) using the ‘Raster Calculator’ tool, creating a raster showing any changes between the two datasets. Locations where scrub had appeared between the two datasets was labelled as Scrub Growth and were coloured green, whereas locations where scrub had disappeared were labelled as Scrub Loss and were coloured red, to allow for easy assessment of scrub loss and growth. The background of the raster was then changed to grey, portraying all areas where there was no change in scrub levels between the most recent and least recent datasets.

#### 2.4.4 Creations of Maps Showing Direction of Changes in Scrub Cover

To identify directional changes in scrub cover, the ground cover raster for the oldest and the most recent dataset for each site (2015 and 2021 for Daneway Banks and 2019 and 2021 for Flatholm Island) were reclassified to give each category of ground cover a unique numerical value, with Grass and Ground vegetation being given a value of 100, Scrub given a value of 10, and Trees a value of 50. The ‘Raster Calculator’ tool was then used to subtract the newer dataset from the older dataset, giving a unique value for each change:

- Change from Ground to Scrub = 100–10 = 90
- Change from Ground to Trees = 100–50 = 50
- Change from Trees to Scrub = 50–10 = 40
- No Change = 0
- Change from Scrub to Trees = 10–50 = −40
- Change from Trees to Ground 50–100 = −50
- Change from Scrub to Ground = 10–100 = −90

These numerical values were then each labelled based on the change that had occurred. The ‘Setnull’ tool was used to remove any data with a value of 0, removing the ‘No Change’ data and leaving a map containing only data on ground cover that changed to or from scrub. The ‘Create Attribute Table’ tool was then used on the datasets to find the total area of each value in square metres.

This was then converted to a percentage by dividing the area of each value by the total area of the site boundary and then multiplying the result by 100.

## 3 Results

### 3.1 Daneway Banks

All 30 points manually measured and confirmed as scrub and ground vegetation during the ground verification carried out during the 2017 and 2021 data collection periods were found to match the height bands assigned to them during the orthomosaic creation, confirming that no erroneous classification had taken place and that any consistent inaccuracies within the data overestimated vegetation height by no more than 1.6 cm, and underestimated vegetation height by no more than 4 cm.

**FIGURE 1:**
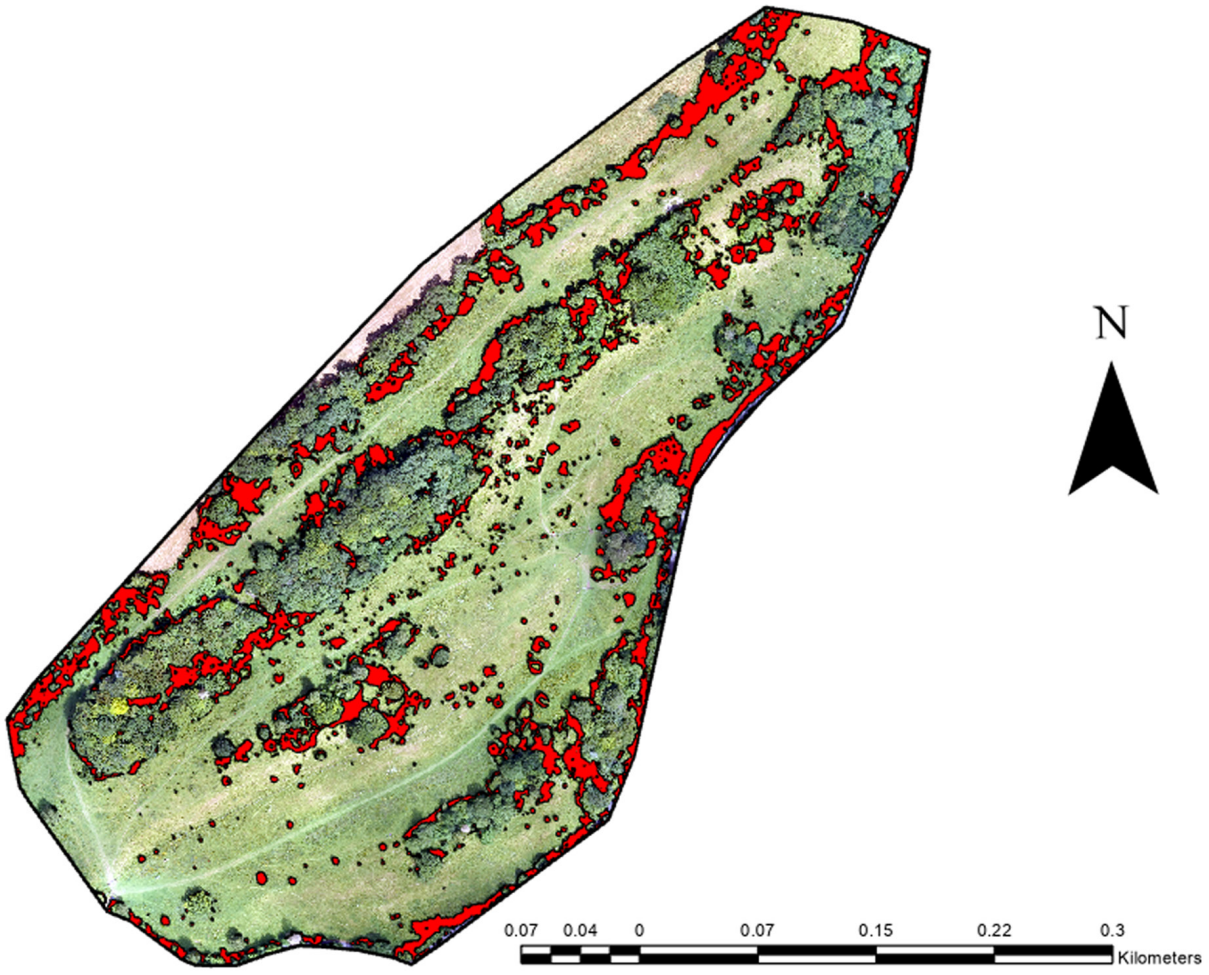
Total scrub cover at Daneway Banks in 2015.

The total area of the Daneway Banks Nature Reserve is 16.927 ha (Natural England 2012) or 169,270 m^2^. For 2015, the on‐site scrub was comprised of 1533 separate stands, with a size range between 0.03 and 2170.35 m^2^. The total area of scrub on the site was 24,763.7 m^2^, comprising 14.63% of the total site (Figure 1).

In 2017, there was a total of 2555 separate stands of scrub, with a size range between 0.01 and 2288.92 m^2^. The total area of scrub on‐site was 27,968 m^2^, making up 16.52% of the total site, a difference of 1.89% and an increase of 12.92% from 2015.

In 2021, there were 2420 separate scrub stands, with a size range between 0.01 and 1556.78 m^2^. The total area of scrub on‐site was 25,200.1 m^2^, making up 14.89% of the total size, a difference of 1.63% and a decrease of 9.87% from 2017, and an increase of 1.78% from 2015.

**FIGURE 2:**
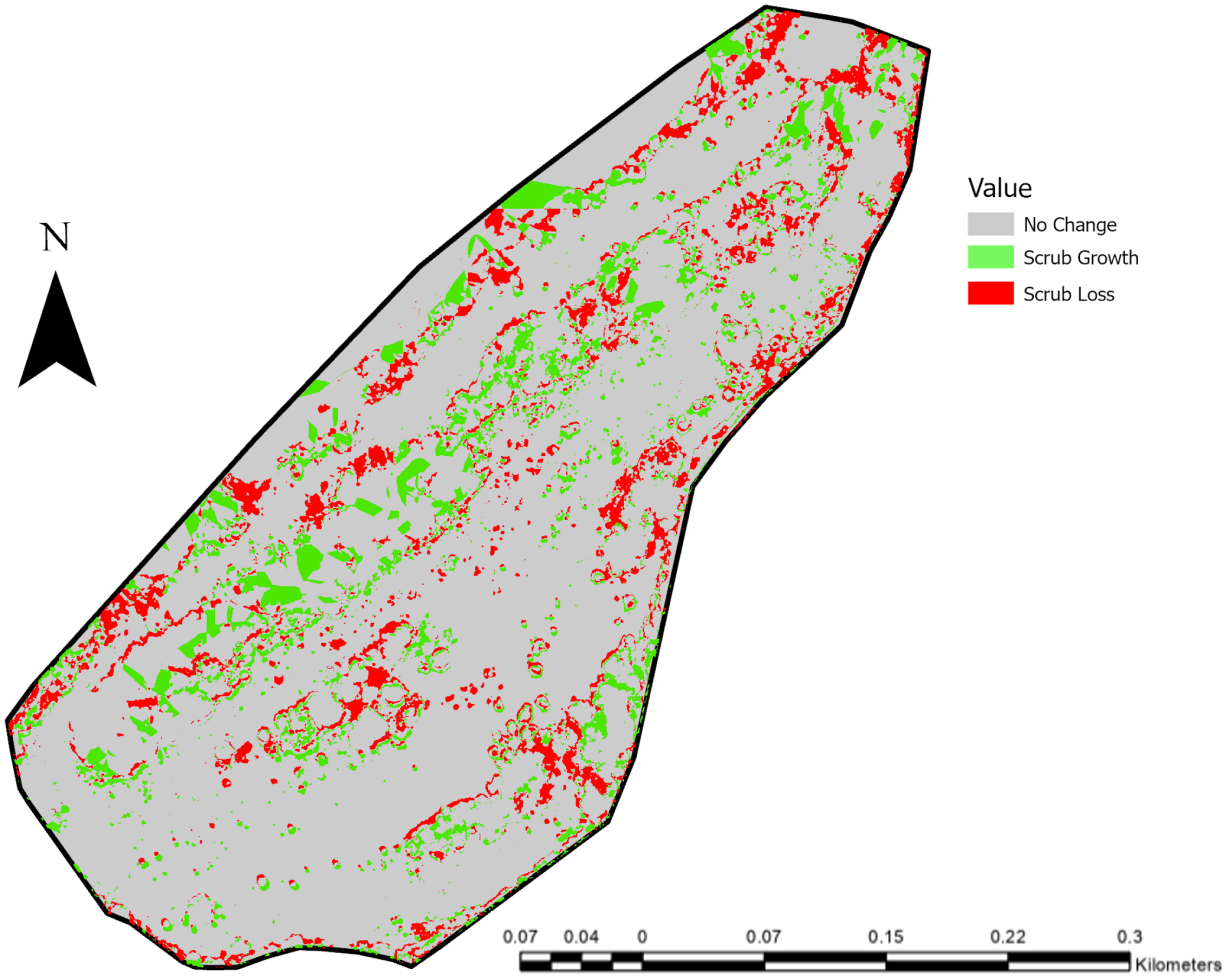
Scrub growth and loss at Daneway Banks between 2015 and 2021.

The total area for scrub growth and loss between 2015 and 2021 were as follows:

- Scrub Growth: 9051.04 m^2^ (5.35% of total area).
- Scrub Loss: 15,875.87 m^2^ (9.38% of total area) (Figure 2).

**FIGURE 3:**
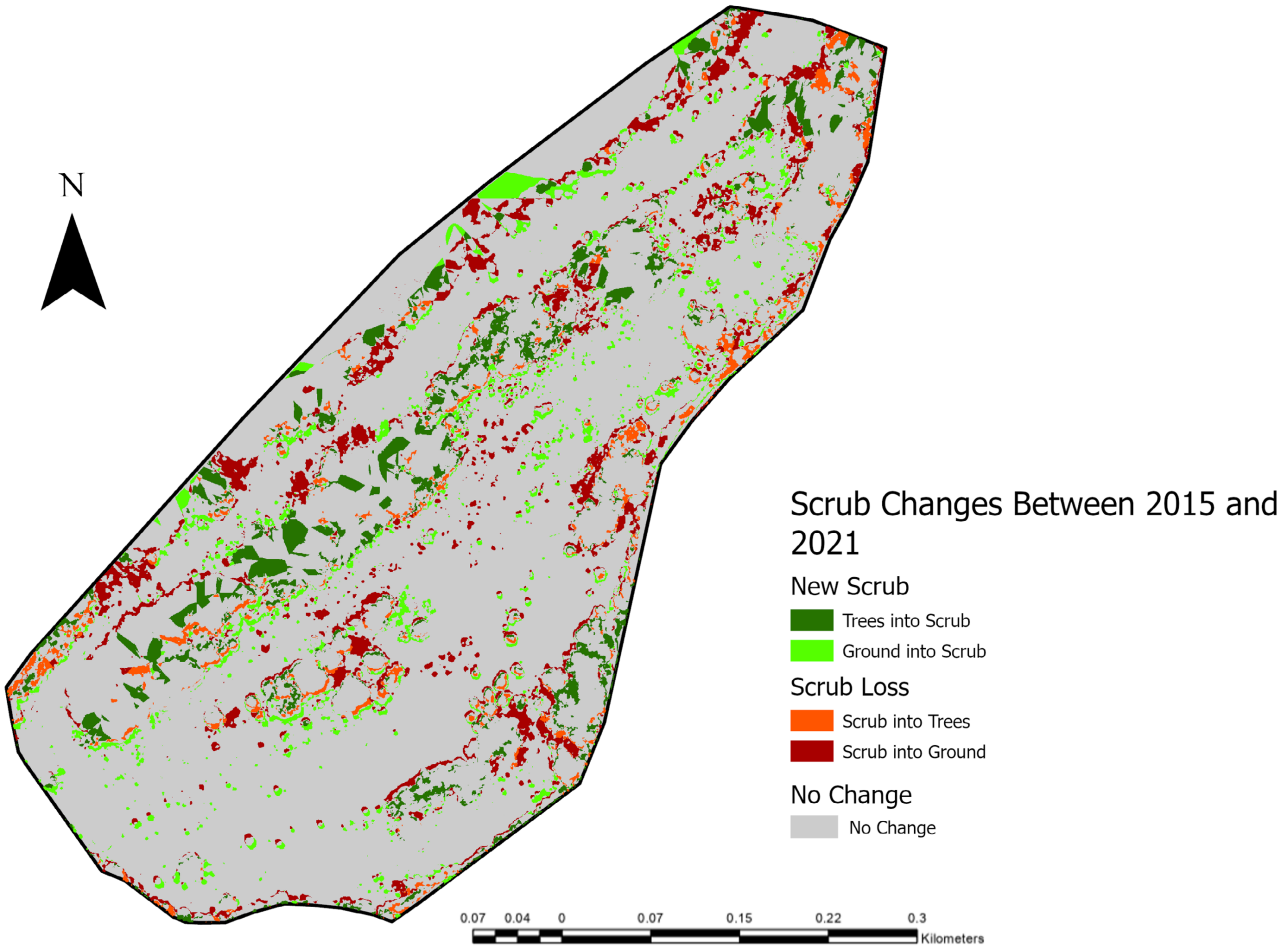
Scrub growth and loss at Daneway Banks between 2015 and 2021 showing directional change.

When looking at the direction of change regarding scrub growth between 2015 and 2021, 57.19% (3.06% of total site area) of new scrub in 2021 was grass or bare ground in 2015, and 42.81% (2.29% of total study area) of new scrub was trees in 2015. In terms of scrub loss between 2015 and 2021, 73.73% (6.92% of total study area) of scrub lost between 2015 and 2021 became grass or bare ground, and 26.27% (2.46% of total study area) of scrub lost between 2015 and 2021 became trees (Figure 3).

### 3.2 Flatholm Island

When determining the study area for Flatholm Island, cliff, and beach habitats, as well as buildings and other functional spaces, were excluded from the study area. Additionally, the northern tip of the island was excluded from the study area, as the UAV flights did not cover it, and the northern part of the island is already heavily managed and contains almost no scrub. The final study area was 167,156.45 m^2^, as opposed to the total terrestrial area of Flatholm, which is approximately 350,000 m^2^.

All 30 points manually measured and confirmed as scrub and ground vegetation during the ground verification carried out during the 2019 and 2021 data collection periods were found to match the height bands assigned to them during the orthomosaic creation, confirming that no erroneous classification had taken place and that any consistent inaccuracies within the data overestimated vegetation height by no more than 0.9 cm, and underestimated vegetation height by no more than 1.5 cm.

**FIGURE 4:**
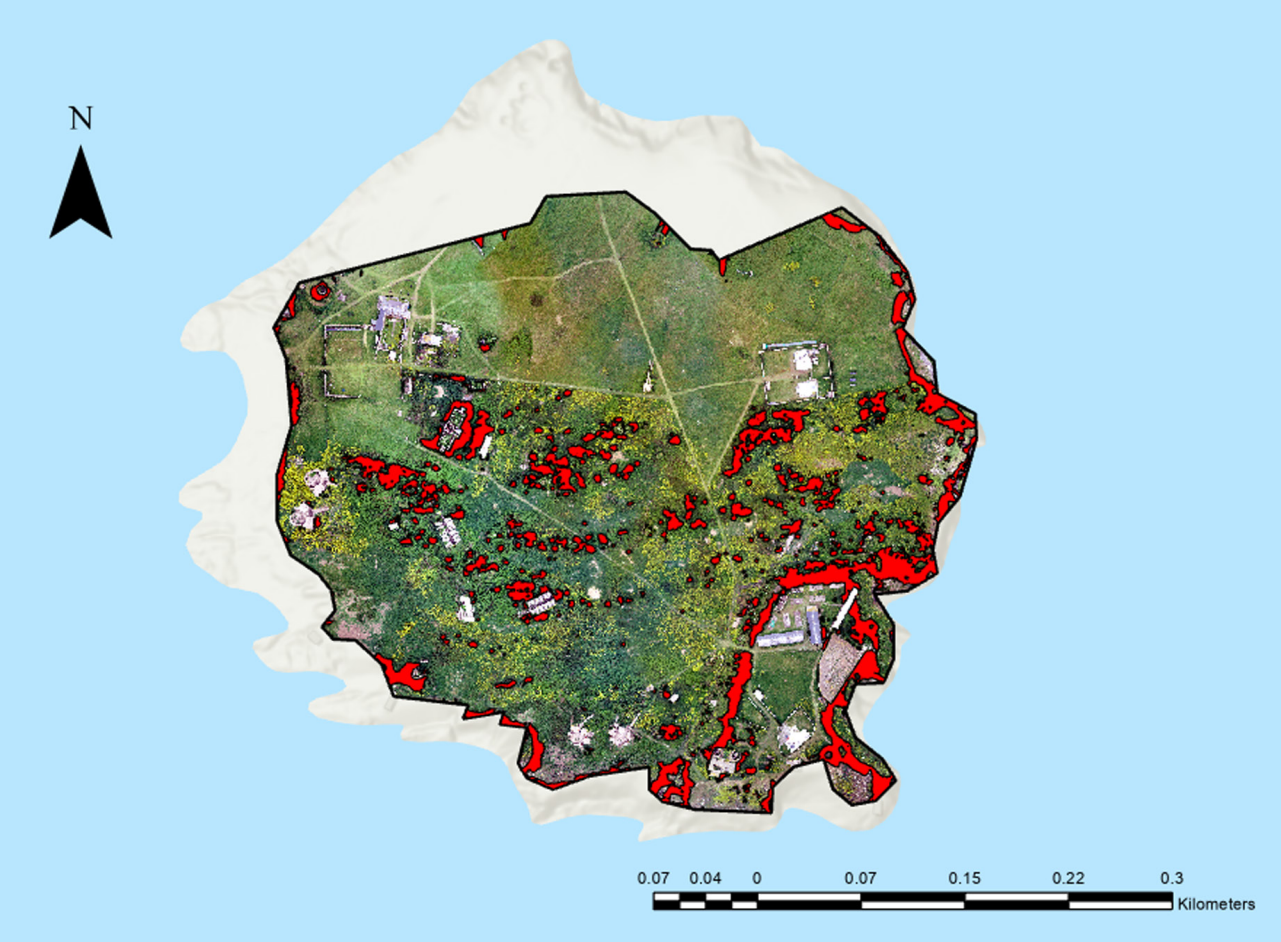
Total scrub cover at the Flatholm Island study site in 2019.

In the 2019 dataset, the on‐site scrub was comprised of 1116 separate stands, with a size range between 0.007 and 2073.34 m^2^. The total area of scrub on the site was 17,018.2 m^2^, comprising 10.18% of the total site (Figure 4).

In 2021, there were 1400 separate scrub stands, with a size range between 0.006 and 752.89 m^2^. The total area of scrub on‐site was 14,560.2 m^2^, making up 8.71% of the total size, a difference of 1.47% and a decrease of 14.44% from 2019.

**FIGURE 5:**
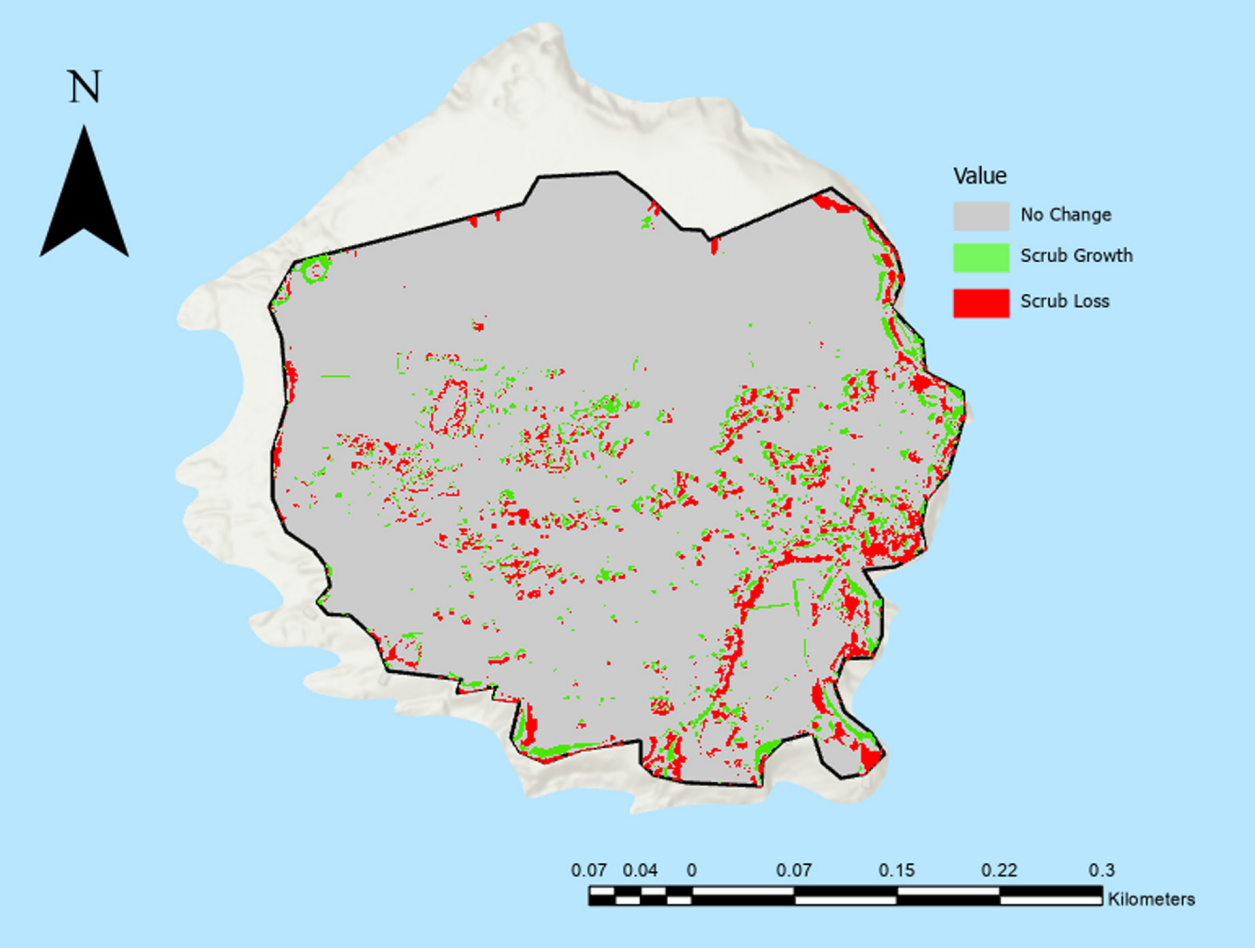
Scrub growth and loss at the Flatholm Island study area between 2019 and 2021.

The total area for scrub growth and loss between 2019 and 2021 were as follows:

- Scrub Growth: 6955.53 m^2^ (4.16% of total area).
- Scrub Loss: 16,935.77 m^2^ (10.13% of total area) (Figure 5).

**FIGURE 6:**
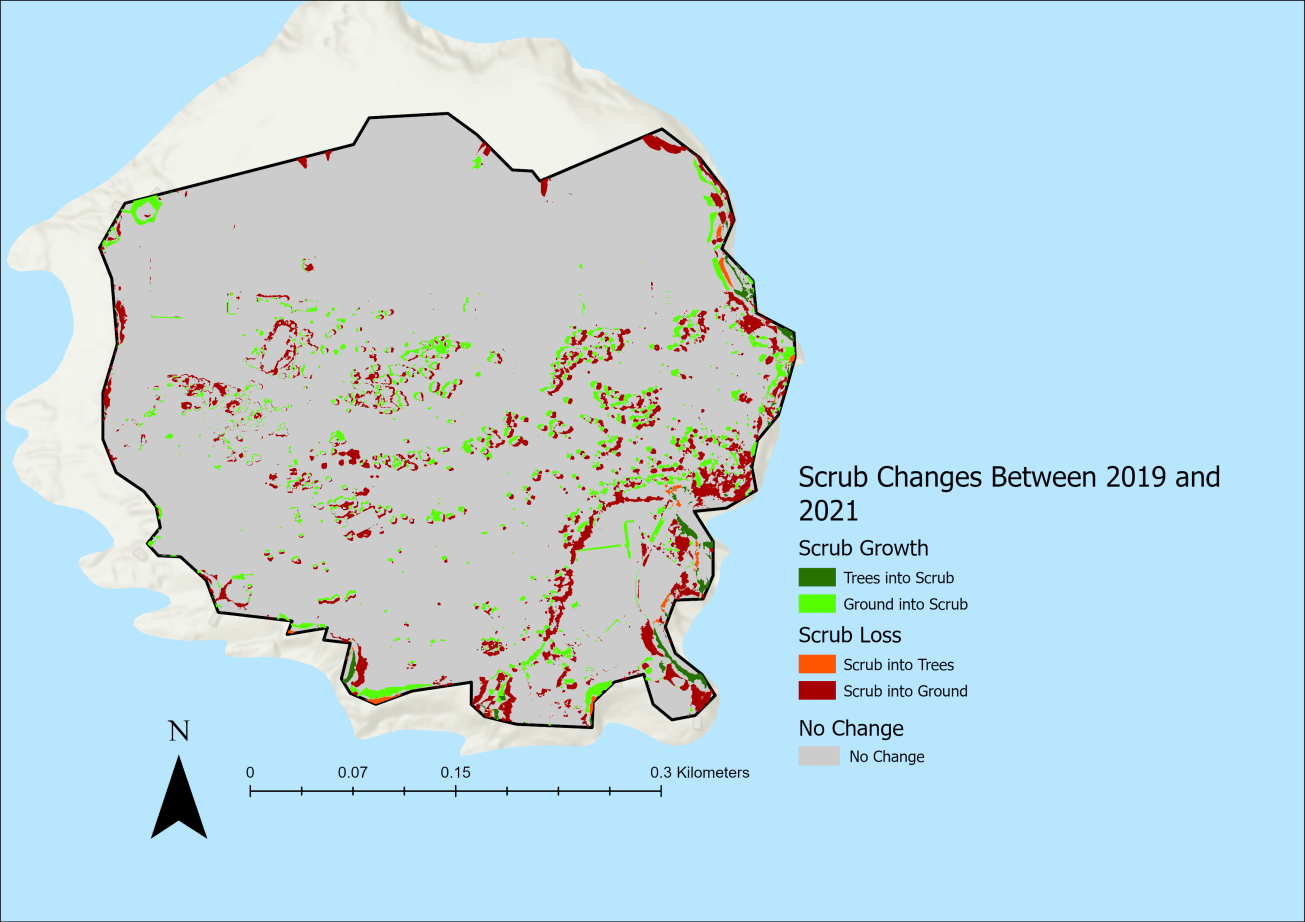
Scrub growth and loss at Flatholm Island between 2019 and 2021 showing directional change.

When looking at the direction of change regarding scrub growth between 2019 and 2021, 88.89% (3.7% of total site area) of new scrub in 2021 was grass or bare ground in 2019, and 11.11% (0.46% of total study area) of new scrub was trees in 2019. In terms of scrub loss between 2019 and 2021, 96.42% (9.77% of total study area) of scrub lost between 2019 and 2021 became grass or bare ground, and 3.58% (0.36% of total study area) of scrub lost between 2019 and 2021 became trees (Figure 6).

## 4 Discussion

This study shows how height maps generated from UAV‐derived aerial images using the method established in Vafidis et al. (2021) can be used to measure temporal and directional change in scrub levels in an accurate, quantified way.

While photogrammetry‐based methods have been used to assess and quantify vegetation structure (Alonzo et al. 2020; Brüllhardt et al. 2020; DiGiacomo et al. 2020), most monitoring of temporal change in vegetation height has been carried out using satellite imagery, with a cell size ranging from 30 m to 1 km (Ghafoor et al. 2022; Li et al. 2022; Zhang et al. 2021; Munsi, Areendran, and Joshi 2012), with most drone‐based monitoring of vegetation height changes using Lidar (De Almeida et al. 2020; Zhou et al. 2020). Very few studies have measured temporal change in vegetation structure using photogrammetry (Nuijten et al. 2021; Watanabe and Kawahara 2016), despite photogrammetry having equivalent accuracy to LiDAR when used to measure vegetation structure (Filippelli, Lefsky, and Rocca 2019). This means that while LiDAR would still be suitable in situations where penetration of the canopy is necessary, photogrammetry as a tool for measuring temporal and directional change in vegetation height works as a cheaper and more accessible alternative to LiDAR‐based methods in situations where canopy penetration is not necessary, while providing more detail than satellite imagery.

**FIGURE 7:**
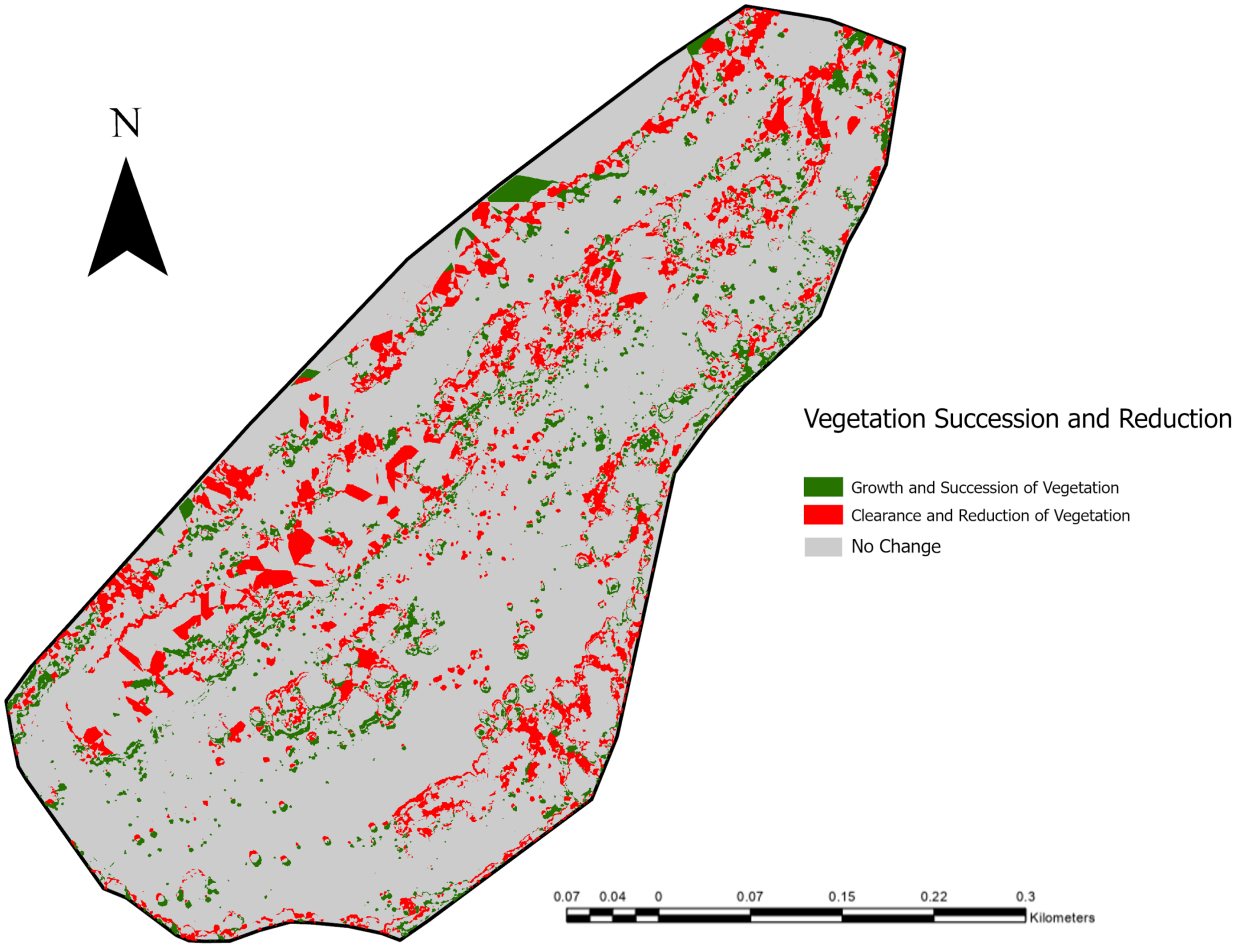
Scrub cover changes at Daneway Banks between 2015 and 2021 categorised into a shift towards vegetation succession or vegetation reduction.

**FIGURE 8:**
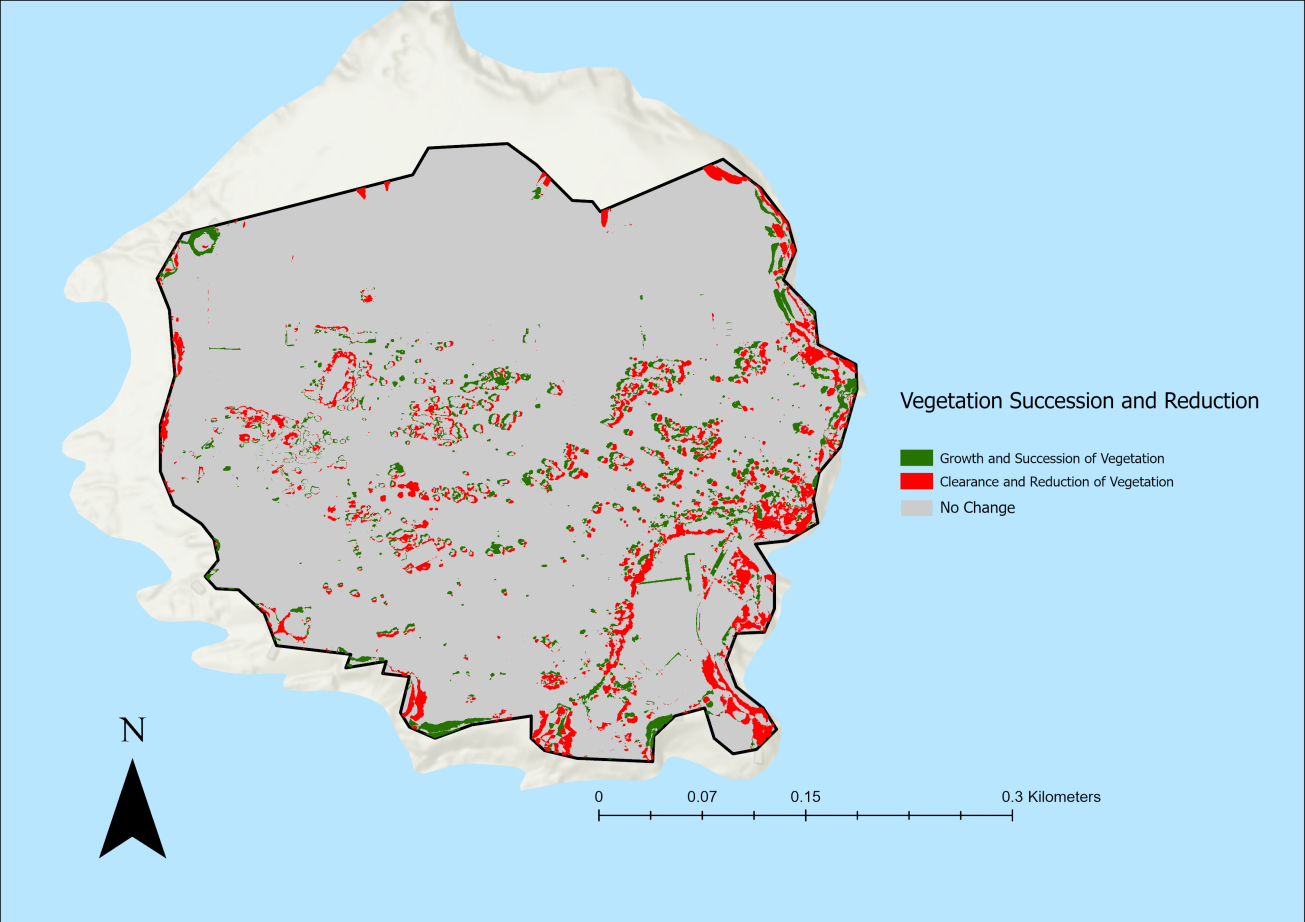
Scrub cover changes at Flatholm Island between 2019 and 2021 categorised into a shift towards vegetation succession or vegetation reduction.

While Flatholm has no specific targets in terms of scrub levels, keeping the site a suitable breeding colony for lesser black‐backed gulls is a conservation priority, with an aim to reduce scrub across the southern half of the island. The directional change at Flatholm Island showed that the majority of scrub loss (96.42%) was due to scrub clearance. In contrast to this, 26.27% of scrub loss at Daneway Banks was due to succession of the scrub into woodland. While this would still be seen as scrub loss, the priority goals of the site are based around the maintaining of grassland habitat and the prevention of succession of grassland, with overall scrub levels kept below 10% (pers. comm) due to the importance of the grassland habitat for bird and invertebrate species. This means that a decrease in scrub due to woodland succession would work against overall site goals, despite still contributing to the specific scrub cover objectives. Because of this, it may be more suitable to view changes in scrub levels as a shift towards either vegetation succession or reduction, with reduction being the management goal. This allows an increase in scrub levels to be beneficial if it is due to tree clearance, as it progresses the site towards a higher level of open habitat. When scrub changes were quantified as a shift towards either vegetation reduction or succession, both sites showed a slight shift towards reduction, with 58.046% of total changes in scrub cover at Daneway Banks (Figure 7), and 60.2% of changes in scrub cover at Flatholm Island favouring a shift towards vegetation reduction (Figure 8).

This shift towards vegetation reduction and the establishment of more open habitat is desirable for both sites, and could be increased if less new scrub was growing in areas that were previously open grassland, meaning that scrub management targets could be met by preventing the growth of new scrub in grassland areas, as opposed to the clearance of existing scrub.

**FIGURE 9:**
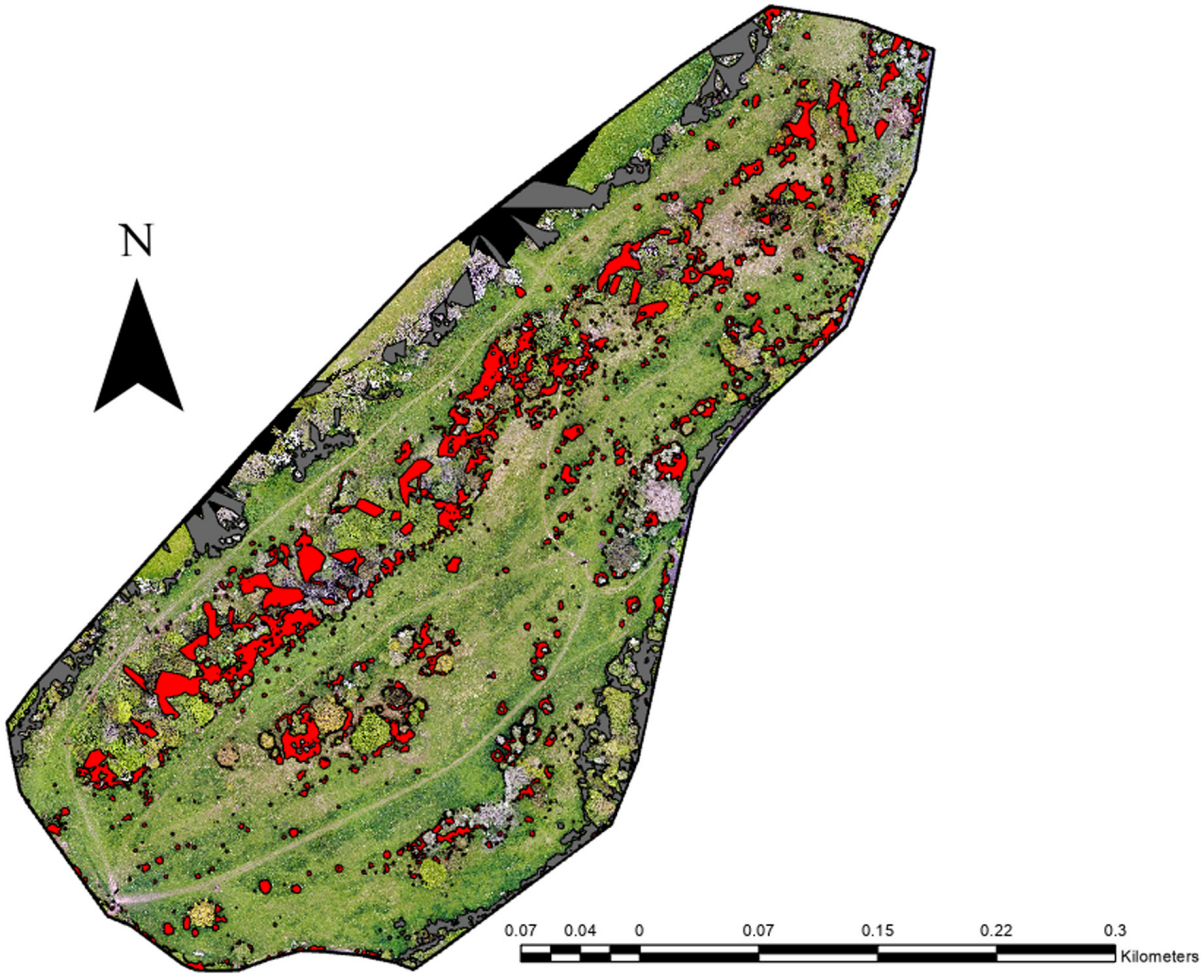
Potential scrub management plan to meet site goals at Daneway banks (Removed scrub coloured grey).

Scrub levels at Daneway Banks increased from 14.63% to 16.52% from 2015 to 2017, before dropping down to 14.89% from 2017 to 2021. While the decrease in scrub levels from 2017 to 2021 shows that progress towards the management target of no more than 10% scrub, or 16.927 m^2^, is ongoing, scrub levels have not dropped below the 2015 levels of 14.63%, and so any management plans will need to be adjusted, with more surveys carried out to ensure that the decline in scrub levels continued. Up to date maps of scrub cover, as well as maps of scrub cover changes could be used to plan the removal of previously‐neglected areas of scrub. For example, the removal of large scrub stands along the northern and southern borders of the site would reduce scrub levels down to 16,749 m^2^, or 9.89% of the site, meeting site management goals, at which point any new scrub stands could be identified and cleared to maintain existing scrub levels (Figure 9).

Flatholm Island scrub levels showed a marked decrease in scrub levels between 2019 and 2021, showing that their current scrub management is sufficient for reducing scrub levels. Assuming that the target scrub levels are similar to Daneway (no more than 10%) then they have achieved this goal and the site management should be changed from decreasing scrub levels to maintaining current levels of scrub. Maps of temporal change in scrub levels could be used to maintain current scrub levels through the identification and removal of new scrub stands as they appear.

However, scrub management goals for both sites are based on ideal levels of scrub estimated using on‐foot surveys, which are highly subjective and often inaccurate (Katzner et al. 2011), and a goal of scrub loss may not be contributing to the overall goal of grassland habitat due to scrub succession into woodland. This means that scrub levels of less than 10% at Daneway Banks may not be a suitable target, as it is based on a subjective, ground‐based assessment of what 10% scrub would look like. Because of this, it may be more suitable to establish new management targets based on photogrammetric point cloud data rather than basing conservation goals on figures collected via inaccurate means. This could then allow for new management goals to be set and monitored more closely with annual surveys.

Although vegetation height has been shown to be an appropriate structural component for the characterisation of scrub (Vafidis et al. 2021; Cunliffe, Brazier, and Anderson 2016), the lack of any other compositional information on the site vegetation is a limitation of the method, particularly in situations where the identification of particular species is required, such as during the identification and removal of invasive species, or the preservation of endangered species. The Orthomosaic maps generated using this method have been shown to be suitable for species identification (Durgan et al. 2020), although ground‐based assessment of species is shown to be more suitable for species with very similar characteristics, or species with specific ground‐based characteristics that are relied on for classification (Hardin et al. 2007). Drones equipped with LiDAR or hyperspectral sensors have been shown to be more effective at identifying individual plant species (Sankey et al. 2018), but these are expensive and may be outside of the budget of most conservation organisations. Whether or not species composition and identification is carried out using the generated orthomosaic, ground assessments or using more advanced sensors will therefore depend on the cost, exact species of note, and how easily identifiable they are.

Another potential limitation is that although the ground verification confirmed that no erroneous classification had taken place and that all manually verified scrub and ground vegetation points were accurately classified, an exact comparison between the height values determined via the ground verification and the drone‐derived height values could have been carried out to determine the exact error values, as it would help to identify any individual errors or outliers (as opposed to the current method of ground verification which only accounted for consistent errors present across the entire dataset), and should be added to any future methodologies. Ground verification points for woodland points could also be obtained in future studies, assuming suitable equipment and training is available to measure the hight of woodland points safely and accurately.

In conclusion, this study demonstrates how height maps of scrub obtained using UAV‐derived imagery can be used to measure temporal and directional changes in scrub levels, as well as identify whether scrub changes are contributing to overall site management goals. This method provides a cheaper and more accessible alternative to LiDAR‐based methods that conservation organisations can carry out with a consumer‐grade drone, facilitating easier monitoring of vegetation structure and scrub levels, especially when combined with more thorough, pre‐existing ground verification methods.

## Author Contributions

**Matthew Jordan:** conceptualization (equal), data curation (equal), formal analysis (lead), investigation (lead), methodology (lead), writing – original draft (lead), writing – review and editing (lead). **Jim Vafidis:** conceptualization (equal), funding acquisition (lead), project administration (lead), supervision (lead). **Mark Steer:** data curation (equal), project administration (equal), supervision (equal), validation (lead). **Kathy Fawcett:** project administration (equal), supervision (equal). **Kathy Meakin:** project administration (equal), resources (equal), supervision (equal). **Gareth Parry:** resources (equal). **Matthew Brown:** resources (equal).

## Conflicts of Interest

The authors declare no conflicts of interest.

## Data Availability

All data collected is included within the article.
